# Supplementary material for: mTORC1 hampers Hedgehog signaling in Tsc2 deficient cells
Source: Life Sci Alliance. 2024 Aug 26;7(11):e202302419. doi: 10.26508/lsa.202302419 (PMC11349048; doi:10.26508/lsa.202302419)
Supplement: Supplementary file 1 [file LSA-2023-02419_TableS1.docx]

Supplementary Tabel 1. Specific significantly P values

| Figure | Experiment | P-value |
| --- | --- | --- |
| Figure 1A | Significant reduced level of *Gli1* mRNA in *Tsc2^-/-^* compared to WT after stimulation with Pur. | 0.0027 |
| Figure 1B | Significant reduced level of *Gli1* mRNA in *Tsc2^-/-^* compared to WT after stimulation with SHh. | 0.015 |
| Figure 1C | Significant increase in *Gli1* mRNA expression in Pur stimulated *Tsc2^-/-^ MEFS* transfected with *pTSC2* compared *to* MOCK transfected cells. | 0.011 |
| Figure 1D | Significant decrease in *Gli1* mRNA expression in Pur stimulated *WT MEFS* transfected with *siTsc2* compared *to* Scramble (Scr) transfected cells. | 0.0075 |
| Figure 2A | Significant increase in *Gli1* mRNA expression in Pur stimulated *Tsc2^-/-^ MEFs* after treatment with Rapa compared to *Tsc2^-/-^*  MEF only stimulated with Pur. | 0.0069 |
| Figure 2B | Significant increase in *Gli1* mRNA expression in SHh stimulated *Tsc2^-/-^ MEFs* after treatment with Rapa compared to *Tsc2^-/-^*  MEF only stimulated with SHh. | 0.0006 |
| Figure 2D | Significant decrease in *Gli1* mRNA expression in *Tsc2^-/-^* and WT *MEFs* after stimulation with SHh and Rapa in combination with Cyclophamine compared to MEFs only stimulated with SHh and Rapa. | *Tsc2^-/-^:* 0.00066  WT: 0.0018 |
| Figure 2E | Significant decrease in *Gli1* mRNA expression in *Tsc2^-/-^* and WT *MEFs* after stimulation with Pur and Rapa in combination with transfection with si*Smo* (+) compared to MEFs transfected with siScr (-). | *Tsc2^-/-^:* 0.0006  WT: 0.0005 |
| Figure 2F | Significant increase in number of ciliated *Tsc2^-/-^ MEFs* after treatment with Rapa compared to untreated *Tsc2^-/-^ MEFs.* | 2.4 x 10^-8^  n>1000 |
| Figure 3A | Significant increase in *Gli1* mRNA expression in Pur stimulated *Tsc2^-/-^ MEFs* after treatment with Rapa compared to untreated cells.  Significant increase in *Gli1* mRNA expression in Pur stimulated *Tsc2^-/-^ MEFs* after treatment with Torin compared to untreated cells. | 0.020  0.020 |
| Figure 3B | Significant increase in *Gli1* mRNA expression in Pur stimulated *Tsc2^-/-^ MEFs* after treatment with Rapa compared to untreated cells.  Significant increase in *Gli1* mRNA expression in Pur stimulated *Tsc2^-/-^ MEFs* after transfection with *simTOR* (+), compared to siScr transfected cells. | 0.0036  0.0099 |
| Figure 3C | Significant increase in *Gli1* mRNA expression in Pur stimulated cells (treated with siSCR, *siRictor or siRptor*) after treatment with Rapa, compared to only Pur stimulated cells  Significant increase in *Gli1* mRNA expression in Pur stimulated *Tsc2^-/-^ MEFs* after transfection with *siRptor*, compared to siScr transfected cells. | 0.0059  0.0058  0.0037  0.0017 |
| Figure 4A | Significant increase in *Gli2* mRNA expression in *Tsc2^-/-^ MEFs* treated with Rapa compared to untreated cells.  Significant increase in *Gli2* mRNA expression in *Tsc2^-/-^ MEFs* treated with Pur and Rapa compared to untreated cells. | 0.045  0.022 |
| Figure 4C | Significant increase in GLI2 protein in WT MEFs treated with Pur compared to untreated cells.  Significant increase in GLI2 protein in WT MEFs stimulated with Pur and Rapa compared to unstimulated cells.  Significant increase in GLI2 protein in *Tsc2^-/-^ MEFs* treated with Pur compared to untreated cells.  Significant increase in GLI2 protein in *Tsc2^-/-^ MEFs* treated with Pur and Rapa compared to untreated and only Rapa treated cells.  (No difference in GLI2 protein in Pur stimulated *Tsc2^-/-^* MEFs treated with Rap compared to only Pur stimulated cells (n=7)). | 0.033  0.021  0.0076  0.034  (0.085) |
| Figure 5A | Significant increase in GLI2 positive cilia in *Tsc2^-/-^* compared to WT in untreated MEFs.  Significant increase in GLI2 positive cilia in Pur stimulated *Tsc2^-/-^* compared to Pur stimulated WT MEFs.  Significant decrease in GLI2 positive cilia in Rapa treated *Tsc2^-/-^* compared to untreated *Tsc2^-/-^* MEFs.  Significant decrease in GLI2 positive cilia in Pur and Rapa treated *Tsc2^-/-^* compared to only Pur treated *Tsc2^-/-^* MEFs.  Significant increase in GLI2 positive cilia in Pur stimulated WT and *Tsc2^-/-^*  MEFs compared to unstimulated MEFs. | 1.56 x10^-14^  2.2x10^-16^  0.026  0.0074  WT and *Tsc2^-/-^*  : 2.2x10^-16^ |
| Figure 6 | Significant increase in *Gli1* mRNA expression in SmoA1 transfected *Tsc2^-/-^ MEFs* treated with Rapa compared to untreated cells.  (No difference in *Gli1* mRNA expression in GLI2ΔN transfected *Tsc2^-/-^ MEFs* treated with Rapa compared to untreated cells). | 0.026  (0.49) |
